# Correlation of Paraoxonase-1 with the Severity of Crohn’s Disease

**DOI:** 10.3390/molecules23102603

**Published:** 2018-10-11

**Authors:** Katarzyna Szczeklik, Tomasz Mach, Dorota Cibor, Danuta Owczarek, Jacek Sapa, Monika Papież, Jolanta Pytko-Polończyk, Wirginia Krzyściak

**Affiliations:** 1 Department of Integrated Dentistry, Jagiellonian University Medical College, Montelupich 4, 31-155 Krakow, Poland; jolanta.pytko-polonczyk@uj.edu.pl; 2 Department of Gastroenterology, Hepatology and Infectious Diseases, Jagiellonian University Medical College, Śniadeckich 5, 31-531 Krakow, Poland; tomasz.mach@uj.edu.pl (T.M.); dorota.cibor@gmail.com (D.C.); owczarek@su.krakow.pl (D.O.); 3 Department of Pharmacological Screening, Jagiellonian University Medical College, 9 Medyczna Street, 30-688 Krakow, Poland; jacek.sapa@uj.edu.pl; 4 Department of Cytobiology, Faculty of Pharmacy, Jagiellonian University Medical College, Medyczna 9, 30-688 Krakow, Poland; monika.papiez@uj.edu.pl; 5 Department of Medical Diagnostics, Faculty of Pharmacy, Jagiellonian University Medical College, Medyczna 9, 30-688 Krakow, Poland; wirginiakrzysciak@cm-uj.krakow.pl

**Keywords:** Crohn’s disease, paraoxonase-1, oxidative stress

## Abstract

Diagnostics of Crohn’s disease (CD) requires noninvasive biomarkers facilitating early detection and differentiation of the disease. Therefore, in this study, we aimed to determine the relationship between paraoxonase-1 (PON-1), the severity of CD, oxidative stress, and inflammation in CD. The CD activity index was based on the current classification. Plasma PON-1 was measured in 47 patients with CD, and in 23 control volunteers. Using quantitative variables such as receiver operating characteristics (ROC) (area under the curve (AUC)), the diagnostic utility of PON-1 in differentiating the severity of CD was assessed. Circulating PON-1 was found to be decreased in the CD group compared to the control group (269.89 vs. 402.56 U/L, respectively), and it correlated well with the disease activity. PON-1 correlated positively with hemoglobin (Hb) (r = 0.539, *p* < 0.001), hematocrit (Ht) (r = 0.48, *p* < 0.001), total cholesterol (TC) (r = 0.343, *p* < 0.001), high density lipoprotein (HDL) (r = 0.536, *p* < 0.001), low density lipoprotein (LDL) (r = 0.54, *p* < 0.001), and triglyceride (TG) (r = 0.561, *p* < 0.001) and correlated negatively with white blood cell count (WBC) (r = −0.262, p = 0.029), platelet count (PLT) (r = −0.326, p = 0.006), C-reactive protein (CRP) (r = −0.61, *p* < 0.001), and malondialdehyde (MDA) (r = −0.924, *p* < 0.001). PON-1 as a marker for CD differentiation possessed a sensitivity and specificity of 93.62% and 91.30%, respectively. CD was found to be associated with the decrease in the levels of PON-1, which correlates well with activity of the disease and reflects the intensification of inflammation, as well as intensified lipid peroxidation. High sensitivity and specificity of PON-1 determines its selection as a good screening test for CD severity.

## 1. Introduction

Crohn’s disease (CD) is classified as a nonspecific inflammatory bowel disease (IBD) [1]. It has multiple pathogenetic factors and it is associated with disorders related to the genes, immune system, environment, and the intestinal microbiota [2]. Moreover, the oxidative processes induced by the free radicals of oxygen have been indicated in the pathophysiology of tissue damage in CD [3]. Considering free radical damage in combination with the increased inflammatory burden, it appears to be of particular importance, as it intensifies tissue damage. It is a common belief that tissue damage in CD results from the improper response of the immune system in the intestinal mucosa against the metabolic products of intestinal bacteria, or other unspecified factors that facilitate a series of inflammatory reactions in the intestine. These changes, commenced by the infiltration of inflammatory cells in the intestinal mucosa, are associated with the release of inflammatory mediators, such as reactive oxygen species (ROS) or reactive nitrogen species (RNS). Both ROS and RNS are proinflammatory factors, which drive each other in CD and inhibit defensive systems (antioxidative enzymes, such as PON-1), resulting in the cellular damage and intensified lipid peroxidation [4]. Malondialdehyde (MDA) is one of well-known end products of lipid peroxidation induced by ROS. Blood PON-1 constitutes an esterase that depends on calcium, which is known to catalyze the hydrolysis of ester bonds, and occurs in the liver, the intestines, or in the plasma, where it binds with high density lipoprotein (HDL) [5]. Most of the PON-1 that is present in the organism is specifically transferred to the HDL molecules, although other lipoprotein fractions such as chylomicrons or very low density lipoprotein (VLDL), are believed to perform the PON-1-to-HDL transport functions [6]. Moreover, it is believed that oxidized lipids are substrates for such activation; hence, PON-1 is associated with antioxidant activity. In addition to the stimulation of nitric oxide synthase in the endothelium, PON-1 mediates independent NO production by the means of HDL, and increases the outflow of cholesterol from macrophages [7]. Therefore, during infection or chronic endotoxemia when the production of macrophages is increased, one can observe a paradoxical decrease in the protective effect of HDL, due to the increased outflow of cholesterol from the stimulated macrophages [8].

It is noteworthy that PON-1 activation is an important process by itself, which is a long-term process associated with the maturation of HDL, a molecule which changes over time due to its interaction with various phospholipid moieties. Hence, the protective role of HDL cannot be easily defined by determining the concentration of total cholesterol HDL (HDL-C) alone, but can be achieved by determining the HDL functionality, which seems to be a better indicator of the observed changes [9]. In fact, several methods have been reported in the literature with respect to the determination of HDL functionality [9]; however, in this study, we have used the method of determining the HDL antioxidant capacity as it is one of the most reliable techniques.

PON-1 is classified among antioxidative enzymes, the activity of which depends on the age of the person; its primary protective role against the ROS comprises the hydrolysis of lipid peroxides [10]. At present, clinical trials are being conducted on PON-1 polymorphism to establish a rapid diagnostic technique and also on establishing a suitable biological therapy for CD. To the best of our knowledge, there is no data regarding the practical uses of PON-1 in CD, and the studies published so far still require confirmation using larger, prospective clinical trials.

“Lipid paradox” in CD consists of elevated risk of cardiovascular complications, despite the low levels of LDL, which suggests that patients with CD might show decreased levels of total cholesterol (TC), low density lipoprotein (LDL), and HDL. According to the paradox, in contrast to the observed decrease in the so-called “bad cholesterol,” such as LDL, an increase in the inflammatory response can be observed, and vice versa, with a decrease in the inflammatory response, there is an increase in the concentration of lipids in the blood [11,12]. This results in an increase in the cardiovascular risk and the reasons for this are still unclear. The relative impact of the autoimmune system on the state of inflammation and dyslipidemia within the risk of cardiovascular in CD are not fully understood, yet an increasing number of studies indicate that patients with lower levels of TC and LDL and lower indices of atherogenicity may increase the risk of cardiovascular diseases [13,14]. Lipid paradoxes associated with low levels of LDL, which in this case have poor prognosis, and high LDL levels, are also observed in patients with advanced heart failure with left ventricular assist device (LVAD) [15]. This shows that inflammation is linked with other factors associated with etiopathogenesis of CD.

According to the literature, inflammation alone is not responsible for the changes within the lipid profile, suggesting that additional mediators participate in the development of CD. Thus, it is reasonable to study other indicators, such as HDL, which is a part of lipid metabolism, and PON-1, which is an antioxidative indicator, or MDA, which is a product of lipid peroxidation. It is noteworthy that changes not only in the HDL levels but also in the HDL–PON-1–MDA association are responsible for the inflammation in the intestine.

In this study, we examined the role of systemic inflammation and lipid peroxidation with respect to the functioning of the antioxidative system as the key components in the development of potential cardiovascular complications in CD. The demonstration of association between C-reactive protein (CRP), MDA, and PON-1 is its expression. This hypothesis appears to be in-line with the concept of accelerated cardiovascular risk and mortality rate with an increase in the inflammatory burden [16,17]. This has been proven in our previous study with respect to the increased levels of lipid peroxidation, suggesting the need to reduce both inflammation and lipid peroxidation [4] in patients with CD.

A similar relationship between low levels of lipids with unfavorable cardiovascular indices has been found in elderly persons, and in patients with rheumatoid arthritis (RA), heart failure (HF), ischemic heart disease (IHD), IBD, or cancer [18,19,20,21,22,23].

Although disorders associated with disturbances in the lipid metabolism [24] and activity of PON-1 [25] have been previously studied with respect to IBD, their association in CD is poorly understood [26]. Therefore, in this study, we aim to extend this knowledge by exploring the association between HDL, PON-1, MDA, and CRP, in CD. In addition, we intend to study the association between the levels of HDL and platelet count (PLT), which may also constitute subclinical image of atherosclerosis in CD. Patients with CD have not been found to exhibit an elevated level of HDL, despite the fact that they exhibit a considerable reduction of PON-1 activity and an increase in the level of lipid peroxidation. Thus, this study might predict the co-occurrence of atherosclerotic complications in patients with CD.

An assessment of prognostic factors of the possible complications is of key importance in making therapeutic decisions. The current strategies aim at obtaining a deep remission of the disease by preventing the possible future surgical procedures or complications, as well as by inhibiting the progress of the disease. In this regard, the sole strategy to treat patients with CD comprises the early immunosuppression or combined treatment of the disease with biological treatment. Thus, the success of the treatment administered is seen as the control of inflammatory response. The modern therapeutic strategies to treat CD have been greatly modified. A personalized approach associated with the use of new disease predictors is needed, which, apart from foreseeing the occurrence of the disease, will be monitoring its course, as well as assessing the efficiency of the treatment administered. It appears that such an approach is increasingly important from the viewpoint of personalized medicine, which constitutes the direction of future studies.

Therefore, in this study, we aimed to determine the association between antioxidative activity in plasma as measured by the level of PON-1, and the severity of CD, as determined with clinical parameters and intensified oxidative stress and elevated inflammation. In addition, we aimed to verify the hypothesis on the prognostic influence of PON-1 on the course and development of possible thromboembolic complications in CD.

## 2. Results

Table 1 presents the characteristics of the group of patients with CD and control volunteers. In terms of age and sex, there were no statistically significant differences in between the examined individuals (*p* = 0.718, χ^2^ test).

The test group demonstrated higher levels of WBC, PLT, CRP, and MDA and lower levels of Hb, Ht, TC, HDL, LDL, and TG as compared to the control group. As presented in Table 1, these values were found to be statistically significant.

**Figure 1:**
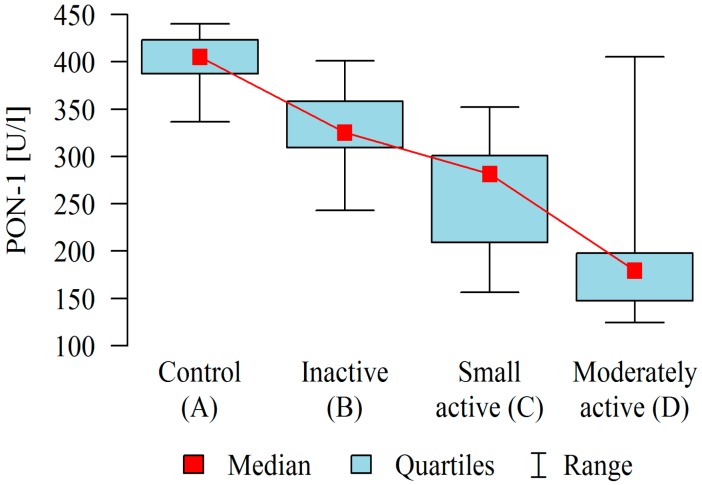
Activity of paraoxonase-1 (PON-1) in the blood plasma of patients in different stages of Crohn’s disease compared to control.

PON-1 activity in the plasma of patients with CD was found to be significantly lower (mean ± SD: 269.89 ± 80.77 U/L) than that of the control group (402.56 ± 27.73 U/L), which was found to change depending on the activity of the disease. As Table 2 and Figure 1 show, the post-hoc analysis demonstrated that in patients with inactive CD, PON-1 activity was statistically and significantly higher than that in patients with moderately active disease.

The results showed a positive correlation between PON-1 and morphological and biochemical parameters: Hb, Ht, TC, HDL, LDL, and TG in patients with CD. We observed that when the PON-1 activity was higher, the levels of the aforementioned parameters were higher. Interestingly, PON-1 significantly and positively correlated (*p* < 0.05) with WBC, PLT, CRP, and with MDA. When the PON-1 activity was found to be higher, the aforementioned parameters found to be in lower levels (Table 3).

**Figure 2:**
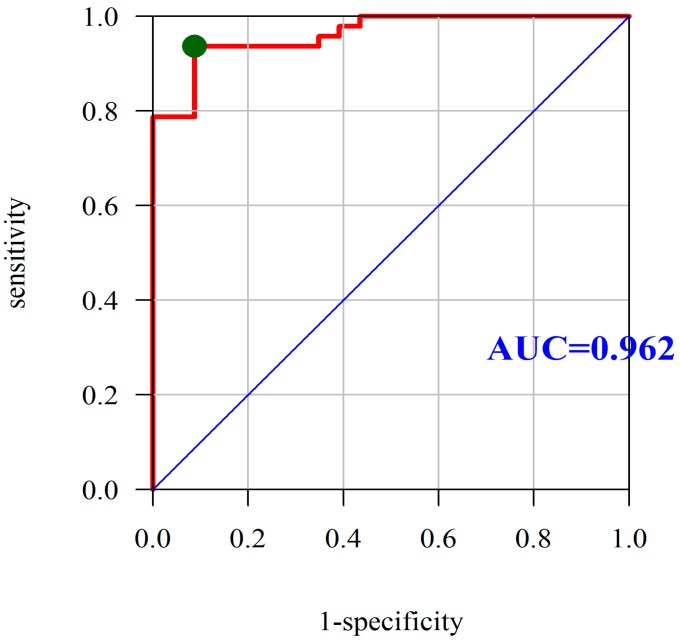
Diagnostic utility of PON-1 was assessed as the predictor of Crohn’s disease based on the analysis of ROC curves. Abbreviations: PON-1, paraoxonase-1; ROC, receiver operating characteristic; AUC, area under the ROC curve; red line mean AUC: area under the ROC curve and green point mean the true cut-off value of the ROC curve.

Based on the analysis of ROC curves, we assessed the diagnostic utility of PON-1 as the predictor of CD by selecting the point located closest to the upper left corner of the chart. Using the area under the ROC curve, we assessed the diagnostic utility of PON-1 as a marker for differentiating the severity of CD. In this case, area under curve (AUC) under the receiver operating characteristic curve (ROC) curve was found to be 0.962, which means that PON-1 is a very good predictor of CD, with an optimum cutoff point equal to 378.25 U/L, and a sensitivity and specificity equal to 93.62% and 91.30%, respectively (Figure 2).

**Figure 3:**
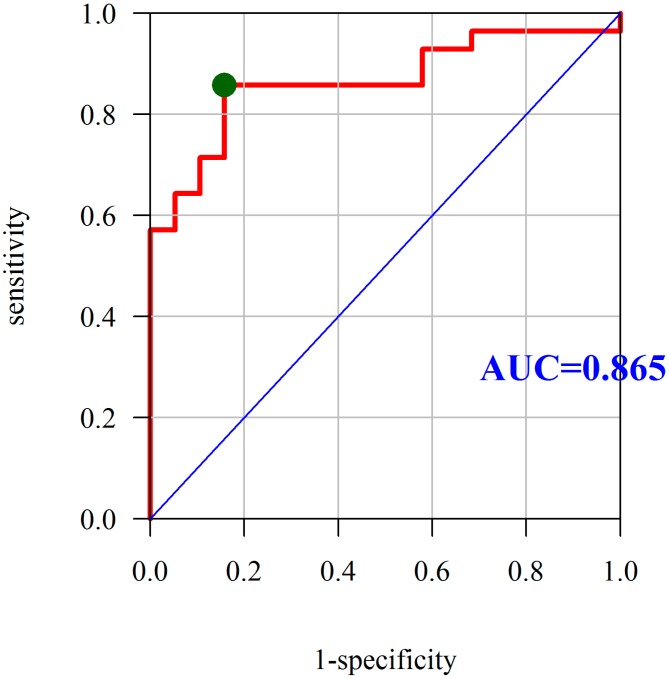
Diagnostic utility of PON-1 was assessed as the predictor of Crohn’s disease activity based on the analysis of ROC curves. Abbreviations: PON-1, paraoxonase-1; ROC, receiver operating characteristic; AUC, area under the ROC curve; red line mean AUC: area under the ROC curve and green point mean the true cut-off value of the ROC curve.

In addition, we observed that PON-1 activity constitutes a very good predictor for the differentiation of CD activity. The AUC under the ROC curve was found to be 0.865, which means that PON-1 is also a good predictor of the active disease. The optimum cutoff point is 305.31 U/L at a sensitivity and specificity of 85.71% and 84.21%, respectively (Figure 3).

## 3. Discussion

Our results show a correlation between the lipid profile, inflammation, and oxidative stress in patients with CD. The mean activity of PON-1 in plasma was found to be statistically and significantly lower in patients with CD than that of control volunteers. In addition, the mean values of Ht, Hb, TC, HDL, LDL, and TG were found to be considerably lower in the group of patients with CD. In contrast, WBC, PLT, CRP, and MDA levels were found to be significantly higher in patients with CD than those of control volunteers.

The results of this study provided the evidence of the role of PON-1 as a potential marker of the severity of the disease. Analysis of the patient subgroups with different degrees of disease progression has indicated a significant difference in the activity of PON-1, in which the lower values indicated active CD.

A lower activity of PON-1 has been observed in various autoimmune diseases such as systemic lupus erythematosus [27], primary and secondary antiphospholipid syndrome [28,29], and functional and structural abnormalities of arteries, such as atherosclerotic lesions [30] or ischemic stroke [31].

In our previous studies, lipid peroxidation, as measured by the level of MDA, was found to be increased, and PON-1 activity was found to be decreased in the patients with CD than that of control volunteers [4]. These results are partially in line with the results obtained by Boehm et al., and Boehm et al., who obtained elevated levels of MDA in patients with CD. On the contrary, their results with respect to PON-1 activity was found to contradict our results. Interestingly, they did not find an association between lipid peroxidation and the activity of the disease [26,32], which has been clearly observed in our study. The results of Maor et al.’s study was in line with ours with respect to observed increased inflammation and oxidative stress and reduced antioxidative status in patients with CD [33].

Another study tested a similar relationship between MDA and PON-1 in patients with RA, and compared the results with control group [34]. Their results did not show any differences in the studied indicators between CD and control groups. In addition, no differences between active and inactive disease in patients with RA were observed. In our study, the elevated production of pro-inflammatory cytokines in the liver (CRP in the CD vs. control group; 13.51 (15.98) vs. 1.26 (1.21) (mg/L), *p* < 0.001 Mann–Whitney test) was found, together with a strong negative correlation between PON-1 and CRP (r = −0.61, *p* < 0.001) (Table 3).

Pro-inflammatory cytokines, including IL-6, induce the synthesis of liver PON-1, but on the contrary, IL-1b and TNF-α inhibit the synthesis of hepatic enzyme [35]. Therapy utilizing anti-TNF-α antibodies in patients with RA induces continuous growth of PON-1 [36]. Thus, patients with RA who are treated with anti-TNF therapy exhibit a decreased prevalence of cardiovascular complications, as compared to other anti-rheumatic drugs. This is probably caused by the effect depending on PON-1 associated with its role in the early uptake of cholesterol and strong antioxidative properties. These properties most likely stem from the enzyme’s capacity to protect LDL as well as HDL [37], against their oxidation. Our study appears to be the first one to demonstrate a negative correlation between PON-1 and MDA (r = −0.924, *p* < 0.001) and a moderate positive correlation between PON-1 and HDL (r = 0.536, *p* < 0.001) and between CDAI and HDL (r = −0.533, *p* < 0.001).

We hypothesize that patient’s age (CD group vs. control: mean ± SD: 35.6 ± 12.69 vs. 34.16 ± 9.84) determines their active compensation mechanisms relative to the increased levels of lipid peroxidation. The impaired antioxidative and anti-atherosclerotic properties of the patient can be assumed to be appearing at a later age, which will be demonstrated by poorer PON-1 function to prevent lipid peroxidation (higher MDA) and decrease in HDL. This will constitute a predictor for the development of atherosclerotic complications [38] in patients with CD. Assessment of long-term survival curves with respect to the test of association between the patient age and PON-1, MDA, and HDL could be an interesting solution for this problem. The disease progression can be assumed to be associated with prolonged inflammation [39,40], which can be seen as elevated mortality due to cardiovascular disorders in patients with CD [16].

The literature provides little evidence for the elevated cardiovascular risk in CD [34,41], but it may be induced by various stimuli, including oxidative stress and chronic inflammation [42]. This might explain the potential CD phenotype and the subclinical phenotype of atherosclerosis in patients with CD. In the plasma of patients with CD, both CRP and MDA levels are found to be elevated, and their values are assumed to grow [43] further with age, as well as in the comorbidities such as atherosclerosis [12]. The deterioration in the coronary flow reserve has been observed with the growth of high sensitivity CRP (hs-CRP), which induces inflammation in the myocarditis or in acute myocardial infarction [44,45]. Variable phenotype as determined by changing PON-1, MDA, or HDL could constitute an added value to determine the risk of cardiovascular complications in patients with CD.

Our observations with respect to the possible thromboembolic complications in CD were confirmed by the increased levels of PLT in the patients with CD as compared to control volunteers (PLT (G/L), mean ± SD: 313.5 ± 116.54 vs. 258.72 ± 45.29). Changes in the aforementioned hemostatic parameters indicate the state of hypercoagulation in patients with CD, even at an inactive stage of the disease. Differences in the levels of PLT count are among the most common factors influencing the development of atherosclerotic lesions [46]. Some concepts of atherosclerosis state platelets as the element joining between vascular hemostasis, congenital immunity, oxidative stress, and inflammation in atherosclerosis [47,48,49].

The increased activity of hemostatic parameters and lowered activity of PON-1 in patients with CD suggest changes in atherosclerosis. In addition, the results obtained have been found to be associated with increased oxidative stress (higher MDA) and increased inflammation (higher CRP) in patients with CD, which supports our hypothesis. In our previous study, we focused our research on increased level of oxidative stress in patients with CD patients [4,32]. Markers of oxidative stress as measured with the level of MDA or other substances reacting with TBARS were found to be considerably elevated in patients with CD. Furthermore, Szczeklik et al. [4] have indicated the predictive role of aforementioned parameters in the determination of the degree of severity of CD (in case of MDA, they obtained an AUC of 0.95 with a cutoff point of >3.82, a sensitivity of 0.93, and a specificity of 0.87). When we compare our previous results of PON-1 to those obtained in this study, we can say that numerous rational arguments exist for the observed differences; one reason could be the elevated synthesis of PON-1 from the liver during increased level of inflammatory responses in patients with CD. In another study, where MDA and PON-1 levels were compared in patients with ulcerative colitis, a decrease in the activity of PON-1 was observed [50], which can be explained by the inhibition of liver enzymes that synthesize PON-1 by the increased level of oxidative stress in the group of patients with CD.

A study indicates that the mechanism of the observed decrease in the activity of PON-1 in the serum of patients with ulcerative colitis is due to the natural capacity of the organism to protect against LDL oxidation, which is accompanied by the deactivation of the enzyme [50]. In addition, an increased level of inactivation of PON-1 was found to be accompanied with an increased level of ROS generation (including superoxide anion) and an increased level of inflammation measured as by elevated IL-1 and TNF-α levels, which may explain the reduced PON-1 activity.

Excessive production of superoxide anion is thought to be associated with the disease, and with aging, it may constitute one of numerous factors that are responsible for the decreased activation of PON-1 in CD via changes in the structure of protein. The conformational change of the PON-1 molecule to HDL and decreased activity of PON-1, due to the conformational changes of PON-1 constituting another possible mechanism. Interestingly, according to our study, the severity of CD as measured by using CDAI has a significant influence on the differences in PON-1 activity. Thus, it can be stated that even in individuals with a low severity of CD, for whom subclinical inflammation is observed during those stages, which are free of clinical symptoms, significant decreases in the activity of PON-1 have been found to be concomitant with the increase of oxidative stress and inflammation. As shown in this study, PON-1, in combination with MDA and CRP, can be a predictor of CD, and more importantly, it can be a predictor of progression or remission of the disease.

Due to the fact that PON-1 may show a different affinity for plasma lipoproteins and it may dissociate in both physiological and pathological conditions, the increased levels of free PON-1 depends on the disease with high levels of oxidative stress [51]. The process of inactivation of PON-1 is associated with various populations of PON-1: HDL–apoA-I-associated PON-1, and the so-called “free” PON-1. The process of inactivation depends on the polymorphic variants of PON-1, including the most commonly described in the literature, namely, 192R/Q polymorphism. RR genotype constitutes HDL–apoA-I-associated PON-1, whereas the QQ and RQ variants are bound with “free” PON-1 (18–46%) [52]. Further research is needed to explain the association between the genotypes of people with different stages of CD and PON-1 activity.

Karban et al., found evidence for the existence of susceptibility to CD depending on the PON-1 and PON-2 polymorphisms. They proved that PON-1 192Q/R polymorphism was significantly less frequent in patients with IBD who showed a higher PON-1 192Q/PON-1 55L/PON2 311S haplotype frequency, and a lower PON-1 192R/PON-1 55L/PON-2 311S haplotype frequency [53]. This suggests that the presence of PON-1 R192 polymorphism in the population of Ashkenazi Jews provides protection against the development of IBD. The association between PON-1 polymorphism and the development and progression of CD seems to be an interesting topic that we intend to verify in our future studies.

There are some limitations of this study. First, we did enroll a large group of patients with CD who were in different stages of the disease. In this study, a total of 14 patients with low severity, 14 patients with moderate severity, and 19 patients with inactive disease were enrolled. Second is the lack of observation with respect to the changes in the activity of PON-1 over time in patients with CD. Third, the results of this study were obtained from patients who visited the clinic for their routine clinical care, and we did not take extreme values of PON-1 and HDL into consideration while accounting for greater clinical instability of these patients. The test group represented only a subset of the total group of patients treated in this and other clinical centers. Fourth, we enrolled patients who expressed their willingness to participate in this study, among only those present at the Hospital Ward. An additional limitation impeding conclusions is with respect to the potential atherosclerotic disorders in patients with CD, which is the absence of atherosclerotic indicators, which would have helped us to correlate the potential biochemical markers of atherosclerosis with the obtained results. Another serious limitation to the study is the relatively young age of patients. Further research that would determine long-term survival curves between patient’s age and PON-1, MDA, and HDL might justify the prophylactic actions undertaken, thereby reducing future risk for the development of cardiovascular complications in patients with CD.

Our results that show a decrease in the activity of PON-1 and a concomitant increase in the values of PLT, CRP, and MDA (significant (*p* < 0.05), and a negative correlation with PLT, CRP, and MDA) may constitute a future predictor of the elevation of atherosclerotic complications in patients with CD. Our study shows that PON-1, as a regulator of lipid metabolism in patients with CD, can fulfill the role of disease severity biomarkers, and moreover, it may prove useful in its prognosis.

The association between the patient’s age and atherosclerotic factors is worthy of a clinical trial, as even in a young patient population, there is a possibility of thromboembolic events to occur, which reduces the efficiency of the treatment administered. This might help to reduce the mortality of patients with CD. Inclusion of the presented biochemical indicators such as PON-1/CRP/MDA/PLT as the potential risk factors for thromboembolic complications may considerably modify previously determined treatment schemes to prevent possible atherosclerotic complications in the future.

We intend to conduct a prospective study that is related to the measurement of PON-1, MDA, and parameters of hemostasis at various time points depending on the clinical condition of patients with CD, in order to confirm the hypothesis associated with the use of PON-1 as a predictor of CD remission. PON-1 in combination with the aforementioned parameters can be used to differentiate between active and inactive forms of CD.

## 4. Materials and Methods

A total of 48 patients with CD (32 men and 26 women; their mean age was 35.6 ± 12.69 years) were recruited in this study. Among them, 19 were in the inactive phase of the disease, 14 exhibited moderate disease activity, and 14 exhibited low disease activity. CD diagnostics was based on radiological tests as confirmed with colonoscopy and histopathological examinations. In each case, laboratory tests were routinely performed, that is, peripheral blood morphology and biochemical tests, including CRP as a short-term prognostic factor. The severity of CD was classified based on the current criteria of Best et al., [54] and Walsh et al., [55]. The severity of CD was classified according to the clinical CDAI, which is a composite scoring system of eight factors based on the selected clinical symptoms. These symptoms were observed by the patient over the past seven days, such as the number of loose/liquid stools per day, the severity of abdominal pain, general well-being, presence of extraintestinal CD manifestations and abdominal mass, use of antidiarrheal drugs, hematocrit level, and change in body weight. Remission of CD (good general well-being and lack of clinical symptoms) was defined as being a CDAI value of below 150. CDAI values above 150 indicated active disease; 150–219 points indicated a mild exacerbation, 220–450 indicates moderate exacerbation, and CDAI values of greater than 450 indicated severe disease [53,54]. CDAI is considered to be the gold standard to assess the activity of CD, and it is applied in clinical practice and in the qualification of patients for the appropriate therapy [55,56]. The other scoring systems are based on endoscopic or histologic assessments of mucosal activity, and they are mainly used in clinical trials to determine a response to therapy.

Patients were treated with azathioprine (2.0–2.5 mg/kg/day), and patients with inflammatory lesions in the colon were additionally treated with mesalamine (2 g/day). Colonoscopy classified patients into the following: exhibiting varying degrees of the severity of the disease (with loss of vessel integrity and/or intestinal wall fragility and/or erosion) and in remission (with normal mucous membrane). The control group comprised 23 persons (12 men and 13 women) without clinical symptoms of the disease; their mean age was 34.16 ± 9.84 years.

PON-1 activity was spectrophotometrically determined in the plasma of 58 patients diagnosed with CD and of 23 healthy persons. In case of patients with CD, 14 of them exhibited moderate disease activity, 14 exhibited mild disease activity, and 19 were in remission.

Prior to the study, we obtained patients’ informed consent. This study was approved by the Bioethics Committee of the Jagiellonian University, and it complied with the Helsinki Declaration.

### 4.1. Blood Samples

All blood samples were obtained in fasting condition during the morning hours between 8:00 and 9:00. The following routine laboratory tests were conducted: complete blood count, and PLT, Hb, and CRP levels were estimated in the hospital laboratory using standard procedures. Plasma concentrations of TC, TG, and HDL were measured enzymatically and analyzed by an automated chemistry analyzer. TC was determined according to the liquid cholesterol method (CHOD/POD); TG was measured by the GPO-PAP method; and LDL cholesterol was calculated according to the Friedewald equation.

Blood samples were centrifuged at 500 *g* for 10 min at room temperature. Plasma samples were collected over morphotic elements and were stored at −80 °C until PON-1 activity was determined.

### 4.2. Theoretical Introduction to the Method

PON-1 activity in blood plasma was determined in accordance with the method described by Eckerson et al., [57] with some modifications. The absorbance of the reaction mixture was recorded spectrophotometrically on a microplate reader (FLUOstar Omega spectrophotometer, BMG Labtech, Ortenberg, Germany). Hydrolysis of paraoxon results in the production of *p*-nitrophenol, which is monitored at a wavelength of 405 nm and at room temperature. Values of kinetic measurements of the enzyme were converted into units per liter (conversion includes the substrate expressed in μmol hydrolyzed to minute per one liter of plasma) using a conversion coefficient. The conversion included data in terms of the standard pathway length of 1 cm, which utilizes the standard molar expiration of the coefficient for *p*-nitrophenol.

### 4.3. Determination of MDA

The concentration of MDA was measured according to the method described by Aust [58]. The assay is based on the fact that MDA as a lipid peroxidation product, which is released from the bonds with amino groups of proteins and other nitrogenous compounds (in an acid environment at about 90 °C) and condenses with two molecules of 2-TBA to form a colored adduct whose maximum absorbance was measured spectrofluorimetrically at the excitation wavelength (Ex) of 536 nm and emission wavelength (Em) of 549 nm in a spectrophotometer (FLUOstar Omega spectrophotometer, BMG Labtech, Germany).

Plasma samples were used in the experiment. The following reagents were purchased from Sigma (Sigma-Aldrich, Poznan, Poland): 3.75% trichloroacetic acid (TCA), 0.025 mol/L HCl, 0.0925% thiobarbituric acid (TBA), and 0.03% butylated hydroxytoluene (BHT). The working solution was freshly prepared daily from a solution containing TBA/TCA/HCl dissolved in water in a ratio of 1:3.

1,1,3,3-tetramethoxypropane was used as a standard at 10–50 nmol/mL concentrations. The test, blank, and reference samples were mixed with the working solution in a ratio of 1:1 (*v*/*v*) (150 μL of the studied sample and 150 μL of the working solution).

The contents of test tubes were mixed for 10 s using a microshaker, and then heated on a boiling water bath for 15 min. After this, the test tubes were instantly cooled on ice for 10 min, and then 1 mL of butanol was added to each test tube. The reaction mixtures were shaken for 30 s. Next, the test tubes were centrifuged for 10 min at 4000 *g* at room temperature, and then 250 µL of the organic layer was carefully transferred to the wells of a black 96-well plate.

### 4.4. Details of Determination

To examine the activity of PON-1, plasma samples that were diluted 10-fold in a dilution buffer (containing 300 μL freshly prepared 1.2 mM paraoxon in 50 mM glycine buffer, containing 1 mM CaCl_2_; pH 10.5) just prior to use and were mixed. After mixing, the samples were incubated for 15 min at 37 °C. To the wells of a 96-microplate, 20 μL of the diluted plasma was admeasured, 200 μL of 1.2 mM paraoxon was added, and the absorbance value was monitored at a wavelength of 405 nm every 15 s for 4 min after prior gentle orbital plate mixing.

Conversion coefficient was determined for OD per minute to units per liter. Results were expressed in international units (Si; U/L): PON-1: OD/min × 11.4 = U/L.

### 4.5. Statistical Analysis

The normality of the distribution of variables was tested using the Shapiro–Wilk test. Comparison of the qualitative variable was performed using a chi-square test (with Yates’ correction for 2 × 2 tables), or a precise Fisher test for low expected values. A comparison of the values of the quantitative variable in two groups was performed using Student’s *t*-test (when a variable had normal distribution in these groups) or a Mann–Whitney test (otherwise).

We compared the values of quantitative variables in three or more groups by using analysis of variance ANOVA (when variable had normal distribution in these groups) or the Kruskal–Wallis test (otherwise). After detecting the statistically significant differences, post hoc analysis was performed with the least significant difference LSD Fisher test (in the case of normality of distribution) or the Dunn test (in the case of absence of normality) to identify statistically and significantly differing groups.

We analyzed the correlations between the qualitative variables by using the Pearson correlation coefficient (when both had normal distribution) or the Spearman correlation coefficient (otherwise). According to Hinkle et al. [59], dependence strength was interpreted as follows: |r| ≥ 0.9—very strong dependence; 0.7 ≤ |r| < 0.9—strong dependence; 0.5 ≤ |r| < 0.7—moderately strong dependence; 0.3 ≤ |r| < 0.5—weak dependence; |r| < 0.3—very weak dependence (negligible).

The cutoff value for PON-1 was established based on the ROC curve, by selecting the point located closest to the left right corner of the chart. Using the area under the ROC curve (AUC), the utility of PON-1 as the predictor was assessed. A significance level of 0.05 was assumed in the analysis. Thus, all values below 0.05 were interpreted as indicating significant relationships. Statistical analysis was performed in R software, version 3.5.0 (Vienna, Austria) [60].

## Figures and Tables

**Table 1:** The characteristics of group of patients with Crohn’s disease (CD) and control volunteers. A *p* value < 0.05 demonstrate significant differences between the two groups.

Features		Control Group		CD Group		*p* *
		*n*	%	*n*	%	
Gender	Women	13	52.00%	26	44.83%	0.718 χ^2^
	Men	12	48.00%	32	55.17%	
**Features**	**Control Group**		**CD Group**			*p* **
	**Mean (SD)**	**Median (quartiles)**	**Mean (SD)**	**Median (quartiles)**		
Age (years)	34.16 (9.84)	30 (26–45)	35.6 (12.69)	34 (26.25–40.75)		0.8 NP
BMI (kg/m^2^)	23.49 (3.26)	22.5 (21.2–25.3)	22.76 (3.35)	22.7 (20.33–24.4)		0.36 P
WBC (G/L)	5.7 (1.09)	5.5 (5–6.7)	7.39 (3.83)	6.88 (5.11–8.06)		0.022 NP
Hb (g/dL)	14.4 (1.37)	14 (13.5–15.2)	12.74 (1.8)	12.95 (11.25–14.2)		<0.001 P
Ht (%)	42.32 (3.35)	41.7 (39.7–43.8)	38.87 (4.39)	38.95 (35.82–41.9)		0.001 NP
PLT (G/L)	258.72 (45.29)	250 (224–285)	313.5 (116.54)	288 (239.25–358.75)		0.048 NP
CRP (mg/L)	1.26 (1.21)	0.76 (0.29–2.41)	13.51 (15.98)	6.87 (2.09–17.75)		<0.001 NP
MDA (nmol/g)	2.88 (0.8)	2.64 (2.25–3.64)	6.83 (3.61)	5.48 (4.17–8.57)		<0.001 NP
TC (mmol/L)	4.73 (0.39)	4.88 (4.47–4.98)	4.35 (0.66)	4.23 (4.05–4.84)		0.005 NP
HDL (mmol/L)	1.14 (0.14)	1.19 (1.02–1.25)	0.87 (0.25)	0.96 (0.62–1.07)		<0.001 NP
LDL (mmol/L)	2.95 (0.38)	2.97 (2.79–3.18)	2.38 (0.49)	2.25 (1.93–2.81)		<0.001 NP
TG (mmol/L)	2.76 (0.27)	2.79 (2.57–2.95)	1.85 (0.31)	1.9 (1.64–2.12)		<0.001 NP

Notes: * χ^2^, chi-square test; ** *p*, normality of distribution (** *p* < 0.01, * *p* < 0.05); parametric analysis; Student’s *t*-test; NP, absence of normality of distribution, nonparametric analysis, Mann–Whitney test. Abbreviations: BMI, body mass index; WBC, white blood cell count; Hb, hemoglobin; Ht, hematocrit; PLT, platelet count; CRP, C-reactive protein; MDA, malondialdehyde; TC, total cholesterol; HDL, high density lipoprotein; LDL, low density lipoprotein; TG, triglyceride.

**Table 2:** Activity of paraoxonase-1 (PON-1) in the plasma of patients with Crohn’s disease (CD) and in control volunteers.

Groups	PON-1 (U/L)								*p* *
	N	Mean	SD	Median	Min	Max	Q1	Q3	
A-Control	23	402.56	27.73	405.31	336.57	440.42	387.59	423.36	<0.001
B-Inactive CD	19	330.47	40.03	325.24	243.13	401.36	309.54	358.16	A > BCD
C-Small active CD	14	262.56	63.22	281.53	156.24	352.15	209.25	301.02	B > D
D-Moderately active CD	14	195.02	73.57	179.38	124.31	405.24	147.51	198.03	

* Absence of normality of distribution in the groups, Kruskal–Wallis test + results of post hoc analysis (Dunn’s test).

**Table 3:** Correlation of paraoxonase-1 (PON-1) activity in plasma of patients with CD and constant variables.

Parameter	Correlation of (PON-1) Activity			
	Correlation Coefficient *	*p*	Dependence	The Power of Dependence
Age (years)	−0.044	0.716	-	-
BMI	0.197	0.103	-	-
WBC	−0.262	0.029	negative	very weak
Hb	0.539	<0.001	positive	average
Ht	0.48	<0.001	positive	weak
PLT	−0.326	0.006	negative	weak
CRP	−0.61	<0.001	negative	average
MDA	−0.924	<0.001	negative	very strong
TC	0.343	0.004	positive	weak
HDL	0.536	<0.001	positive	average
LDL	0.54	<0.001	positive	average
TG	0.561	<0.001	positive	average

Notes: * Absence of normality of distribution of at least one of the correlated variables, Spearman correlation coefficient. Abbreviations: WBC, white blood cell count; Hb, hemoglobin; Ht, hematocrit; PLT, platelet count; CRP, C-reactive protein; MDA, malondialdehyde; TC, total cholesterol; HDL, high density lipoprotein; LDL, low density lipoprotein; TG, triglyceride.

## Abbreviations

AUC	area under the ROC curve
BHT	butylated hydroxytoluene
CD	Crohn’s disease
CDAI	Crohn’s disease activity index
CHOD/POD method	liquid cholesterol method
CRP	C-reactive protein
Hb	hemoglobin
HDL	high-density lipoprotein
HF	heart failure
Ht	hematocrit
IBD	inflammatory bowel disease
IHD	ischemic heart disease
IL	interleukin
LDL	low-density lipoprotein
LVAD	left ventricular assist device
MDA	malondialdehyde
PLT	platelet count
PON-1	paraoxonase-1
RA	rheumatoid arthritis
RNS	reactive nitrogen species
ROS	reactive oxygen species
TBA	thiobarbituric acid
TBARS	thiobarbituric acid reactive substances
TC	total cholesterol
TCA	trichloroacetic acid
TG	triglyceride
TNF-α	tumor necrosis factor
VLDL	very low density lipoprotein
WBC	white blood cell count
